# Prognostic Significance of Thyroglobulin Antibodies in Differentiated Thyroid Cancer

**DOI:** 10.1155/2020/8312628

**Published:** 2020-04-14

**Authors:** Jordi L. Reverter, Irene Rosas-Allende, Carlos Puig-Jove, Carles Zafon, Ana Megia, Ignasi Castells, Eduarda Pizarro, Manel Puig-Domingo, M. Luisa Granada

**Affiliations:** ^1^Departments of Endocrinology and Nutrition, Germans Trias i Pujol Hospital and Research Institute, Universitat Autònoma de Barcelona, CIBERER (ISCIII), Barcelona, Spain; ^2^Consortium for the Study of Thyroid Cancer, CECAT, Barcelona, Spain; ^3^Clinical Biochemistry, Germans Trias i Pujol Hospital and Research Institute, Universitat Autònoma de Barcelona, CIBERER (ISCIII), Barcelona, Spain; ^4^Department of Endocrinology, Hospital Vall d'Hebron, Diabetes and Metabolism Research Unit, Vall d'Hebron Institut de Recerca, Universitat Autònoma de Barcelona, CIBERDEM (ISCIII), Barcelona, Spain; ^5^Department of Endocrinology and Nutrition, Hospital Universitari Joan XXIII, Research Institute Pere Virgili, CIBERDEM (ISCIII), Tarragona, Spain; ^6^Endocrinology Service, Hospital General de Granollers, Barcelona, Spain; ^7^Endocrinology Service, Hospital de Mataró, Barcelona, Spain

## Abstract

**Objective:**

To investigate whether variations in thyroglobulin autoantibodies (TgAb) are related to the recurrence or persistence of differentiated thyroid carcinoma (DTC) and may therefore be useful as surrogate tumor markers. *Design and Methods*. We retrospectively studied 98 subjects (83 women, 47 ± 15 years old) from an initial cohort of 1017 patients treated for DTC in five hospitals, with positive TgAb at any time during the follow-up. Patients presented five different patterns of evolution of serum TgAb concentrations: (1) stable positive TgAb, (2) *de novo* appearance, (3) an increase of more than 50%, (4) TgAb levels from positive to negative, and (5) a decrease of more than 50%.

**Results:**

In the group of 11 patients with stable TgAb, four cases presented persistence of the disease with structural incomplete response. In the group of 22 patients with sustained increasing trend rising more than 50% or *de novo* detectable TgAb levels, three patients were diagnosed with structural incomplete response. There was no evidence of recurrence or persistence of the disease in any of the 65 patients who showed a significant decrease in (*n* = 35) or disappearance of (*n* = 30) TgAb.

**Conclusions:**

Our results suggest that not only the appearance of a significant increase in TgAb but also stable concentrations of TgAb should be regarded as a sufficient risk condition for an active search for recurrent or persistent disease. Conversely, a significant decrease in TgAb levels can represent a good prognostic sign.

## 1. Introduction

Differentiated thyroid carcinoma (DTC) has an excellent survival rate. Despite this, recurrences occur in 5%–20% of patients, and because of its prevalence, DTC is the leading cause of death among all endocrine tumors [1]. Serum thyroglobulin (Tg) is produced and secreted exclusively by benign or differentiated malignant thyroid cells, and it is considered a highly sensitive and specific tumor marker for patients with DTC after removal of benign and malignant thyroid tissue by surgery and ^131^I ablation [2]. Thyroglobulin is usually measured by noncompetitive automated immunoassays using two monoclonal antibodies directed against two epitopes of the molecule. Over the years, advances in assay technologies have led to important improvements in the analytical performances of Tg immunometric assays (IMAs), with the sensitivity of detection increasing tenfold from 0.5–1.0 *μ*g/L in the first-generation IMAs to 0.05–0.10 *μ*g/L in the second-generation IMAs. However, the main limitation of determination of Tg is that it can be modified by serum Tg autoantibodies (TgAb), which give falsely decreased results in IMA assays. Furthermore, this interference is variable among patients and only correlates with TgAb concentrations [3]. Therefore, the degree of interference cannot be predicted and an accurate Tg value cannot be obtained. It should also be noted that any TgAb assay will detect only a subset of interfering TgAbs [4].

Serum TgAb are present in about 60% of patients with autoimmune thyroid disease and are more frequent in females [5]. It has been reported that approximately 20%–25% of patients are with DTC test positive for TgAb, depending on the specific assay used and the study population [6]. In patients with DTC who test positive for TgAb, an undetectable Tg cannot be judged as the absence of disease and such cases are classified as “indeterminate response” according to the American Thyroid Association (ATA) guidelines [7]. In these patients, it has been suggested that the trend towards increasing TgAb concentration could reflect changes in thyroid tissue mass [8]. The rationale behind TgAb measurement and the interpretation of results in DTC patients is based on the theory that the presence of an antigenic stimulus induced by Tg is what drives TgAb production [9].

Various authors have suggested that TgAb can be used as a surrogate tumor marker for disease recurrence during long-term follow-up in DTC [10–13]. However, this is a controversial issue, mainly because the presence of TgAb may not be useful in a significant proportion of patients due its low prevalence [14], and also because there is as yet no clear definition of the magnitude of TgAb level variation and the period of time over which it occurs that would be necessary for it to be regarded as a significant trend in their concentrations.

The aim of this study was to investigate the prevalence of TgAb in a large cohort of DTC patients and its potential usefulness as a surrogate tumor marker by evaluating whether variations in TgAb levels are associated with disease activity.

## 2. Materials and Methods

### 2.1. Patients

We retrospectively studied patients treated for DTC between 2010 and 2016 at the Endocrinology Departments of five hospitals in the region of Catalonia, Spain: the Germans Trias i Pujol University Hospital in Badalona, the Vall d'Hebron University Hospital in Barcelona, the Joan XXIII Hospital in Tarragona, the Granollers General Hospital in Granollers, and the Mataró Hospital in Mataró. Patients were treated with complete thyroidectomy, additional lymph node dissection if indicated, suppressive levothyroxine treatment, and postoperative remnant ablation with ^131^I according to ATA guidelines [7]. Laboratory evaluation consisted of taking measurements of serum Tg, TgAb, and TSH at least once every year. Recurrence was defined as detection of structural disease by ultrasound or other image explorations, whole-body scan, or presence of malignant cells in a fine-needle aspiration biopsy or in tissue obtained during reoperation. Patient characteristics (gender and age), laboratory measurements (TSH, Tg, and TgAb levels), tumor characteristics (histological subtype: papillary or follicular), and data regarding disease status during follow-up and duration of follow-up were also recorded. All patients with at least one year of follow-up and at least two determinations of Tg and TgAb, measured in a period of at least 6 months, were included in this study. Patients with TgAb levels below functional sensitivity of the assay in all the samples obtained during the follow-up were excluded.

The study was conducted in accordance with the Declaration of Helsinki and was approved by the local Human Research Ethics Committee of the Hospital Germans Trias i Pujol. Consent was obtained from each patient after full explanation of the purpose and nature of all procedures used.

### 2.2. Laboratory Methods

Thyroglobulin determinations were carried out by Elecsys® electro chemiluminescent electromagnetic immunoassay (ICMA) (Roche Diagnostics). TgAb were measured by chemiluminescent immunoassay using either the Liaison® system (DiaSorin, Saluggia, Italy) in one of the participating centers and the Immulite® 2000 XPi analyzer (Siemens Healthcare Diagnostics, Llanberis, UK) in the others. Functional sensitivity (FS), defined as the lowest TgAb concentration that can be measured with an interassay coefficient of variation <20%, was 10 UI/mL. A result was considered positive for TgAb if serum levels were ≥10 IU/mL (above FS) in samples measured by the Liaison® and ≥20 IU/mL in those measured with the Immulite® 2000 XPi.

Given that TgAb may rise transiently after surgery and ^131^I ablation therapy, TgAb were measured at least 6 months after these treatment procedures.

Based on TgAb presence and changes in serum TgAb concentrations, patients presented one of the five different patterns: (1) stable positive TgAb concentrations with variations less than 50% throughout follow-up, (2) increased levels of TgAb above functional sensitivity (*de novo* appearance from negative to positive), (3) sustained increasing trend (increasing more than 50% in levels), (4) TgAb levels decreasing below FS with a shift in TgAb status from positive to persistently negative, or (5) levels of TgAb decreasing more than 50%.

### 2.3. Imaging Modalities

Neck ultrasound was performed at least yearly, and further imaging techniques (^131^Iodine, CT scan, MRI, 18-FDG PET/CT) were used if indicated.

### 2.4. Recurrent or Persistent Disease

Recurrence or persistence of the disease had to be proven by cytology, histology, or positive post-^131^I therapy scan. In this study, the ATA management guidelines criteria for recurrence and persistence of the disease were used [7].

### 2.5. Statistical Analysis

Descriptive statistics were applied to all collected variables expressed as frequencies for categorical data or mean values ± standard deviations for continuous data. Group comparisons were carried out using the chi-square test. A *p* value less than 0.05 was considered significant. Statistical analysis was performed using the IBM SPSS® Statistics 24 software package (IBM Corp., Armonk, NY, USA).

## 3. Results

### 3.1. Patients

**Figure 1:**
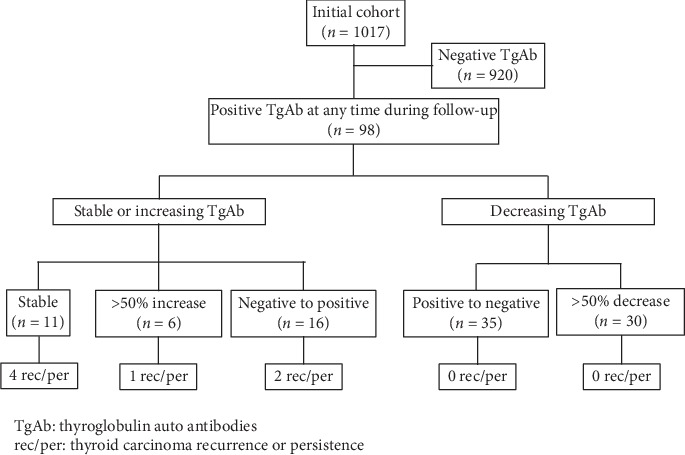
Flow chart of the study population and results.

From an initial cohort of 1017 patients with DTC, 98 (*n* = 83 females, 47 ± 15 y), representing 9.5%, presented positive TgAb at some point during follow-up.  Table 1 shows the characteristics of these patients. This group included papillary thyroid cancer (*n* = 86) and follicular thyroid carcinoma (*n* = 12). Patient's initial Tg levels were 0.75 ± 1.3 ng/mL. In terms of TgAb status, 11 patients had stable positive TgAb concentrations, 16 patients had *de novo* appearance, and 6 patients had a significant increase in TgAb. On the other hand, in 65 patients, TgAb levels decreased above FS (*n* = 35) or >50% (*n* = 30) during follow-up.  Figure 1 represents a flowchart of the results.

### 3.2. Relationship between TgAb and Recurrence or Persistence of the Disease

Table 2 shows the characteristics of the 7 patients with disease recurrence according to TgAb levels during the follow-up. In the group of 11 patients with stable TgAb, the mean follow-up period was 78.9 ± 9.0 months, and four cases presented persistence of the disease with structural incomplete response. In only one of the 6 patients with sustained increasing trend rising more than 50% was recurrence detected. Sixteen patients presented *de novo* detectable TgAb levels and two of these patients were diagnosed with structural incomplete response. The mean follow-up period in the group of increasing or *de novo* appearance TgAb was 75.7 ± 10.1 months.

There was no evidence of recurrence or persistence of the disease in any of the 65 patients who showed a significant decrease in (*n* = 35) or disappearance of (*n* = 30) TgAb after a mean follow-up of 65 ± 10.9 months.

When patients with pattern type 1 (increase) were compared with those in pattern type 2 (decrease), no significant differences were observed regarding persistence or recurrence of the disease (*p* < 0.2). The relative risk of having active disease was higher but not significantly different in patients with stable TgAb with respect to those with *de novo* appearance (*p*=0.3) or a significant increase (*p*=0.2). Patients with positive TgAb presented a highly significant RR of persistent or recurrent disease (not quantifiable due to the absence of cases with active disease in the group of patients whose TgAb decreased significantly or disappeared).

## 4. Discussion

First, in this study involving a relatively large cohort of patients with DTC and positive TgAb at some point during follow-up, the prevalence of positive TgAb was relatively low, less than 10%, with respect to previous reports [14–16]. Second, a decrease in TgAb to undetectable levels below the FS of the method or a significant decrease >50% identified those cases with no evidence of disease recurrence or persistence; this group was strongly represented as it corresponded to 65 out of the 98 patients. And finally, the cases with active disease were detected in patients with stable levels of a significant increase in or *de novo* appearance of TgAb, thus supporting the usefulness of the measurement of TgAb as a surrogate marker of active disease in DTC [17].

From a clinical point of view, these results suggest that only a minority of cases of DTC show positive TgAb during follow-up. This low prevalence of TgAb, together with the fact that in more than half of cases, TgAb disappear or decrease during follow-up, raises the question of whether its measurement would be of major utility in daily clinical practice. The prevalence reported in various studies on this topic is not only higher (25%) than in the general population (10%) but also higher than that seen in our results [4, 16]. Based on our data, the actual impact on DTC populations may be limited. As the prevalence of positive TgAb in our DTC population was 10% and the probability of recurrence of disease in papillary thyroid carcinoma (PTC) has been reported to reach 20% at some point during the patient's lifetime, the combined probability of recurrence in patients with positive TgAb is 2 out of every 100 DTC patients. In connection with this, two long-term retrospective studies, covering follow-up periods of 12 to 29 years, detected only 2.5 patients per year with positive TgAb [11, 18]. Population factors may be at the root of this difference in TgAb prevalence [19].

Independent of its prevalence, in those patients showing positive TgAb, who are considered a diagnostic challenge, serum levels of TgAb have been proposed as a surrogate tumor marker [10, 11, 15, 16]. Our results are in accordance with the ATA guidelines statement [7], which indicates that a decline in TgAb levels over time is considered a good prognostic sign [20], while rising antibody levels, in the absence of an acute injury to the thyroid, significantly increase the risk of persistent or recurrent thyroid cancer [21]. Specifically, recurrent or progressive disease should be suspected in those patients initially positive for TgAb that then become negative but subsequently show rising levels of TgAb [7]. In our study, all patients with persistent or recurrent disease had *de novo* appearance of TgAb or a significant >50% increase during follow-up, but also no active DTC was observed in any patient in whom TgAb levels became undetectable or decreased significantly over time. These results agree with a previous prospective study of 576 patients with DTC, which found no statistically significant difference in recurrence development between patients with persistent undetectable TgAb (2.6%) compared with those with borderline TgAb (3.2%) during follow-up. However, patients with borderline TgAb were more likely to progress to elevated TgAb, and this event was significantly associated with recurrence [22].

The interference of TgAb in Tg determinations is an issue that has not yet been resolved in the clinical assessment of thyroid carcinoma patients classified as “undetermined response.” In the absence of alternative methods, neck ultrasound and other image explorations have become the basis of risk stratification [7]. Some studies have proposed that TgAb persistence suggests persistence or recurrence of the disease [20, 21] even when limiting the time frame to the first year after treatment in TgAb positive PTC patients [23]. Our study does not support these findings. In our cohort, the pattern depicting the persistence of stable levels or increase in TgAb throughout follow-up is associated with active disease. However, other studies have found no correlation between persistent TgAb and higher frequency of recurrence or mortality [24].

An important point to consider is the need to establish clear definitions for trends in TgAb levels and the level at which the TgAb is considered as positive. In this regard, reports of the cutoff point of serum TgAb levels are scarce and inconsistent [25]. Some authors have used the reference range provided by the manufacturer as a limit, but this specific value was established to identify autoimmune thyroid disease and not to define interference with Tg [16]. We consider, according to other authors [11], that TgAb levels “positive” or “negative” depend on the functional sensitivity of the assay since interference can occur at detectable TgAb concentrations below the reference limit (borderline TgAb). In addition, the quantification of TgAb levels allows the evaluation of variations over time, which is also very important for defining the trend pattern of an individual patient [8]. Some of the reasons for the discrepancies between studies may be related to some selection bias or differences in study design.

Another potential source of discrepancy is the two different methods used to analyze TgAb in our study. In this sense, despite being standardized according to the same World Health Organization International Standard 65/93, these two tests give different numerical results. To validate our results, we made a previous comparison of the results obtained by these two trials in a sample of 80 subjects, showing that they had a high level of correlation (*r* = 0.92, *p* < 0.001) with proportional differences (slope = 0.70), which indicates that the results obtained by Immulite (Siemens) were consistently higher than those obtained with Liason (DiaSorin) (data not shown).

The presence of endogenous TgAb may interfere in Tg measurements, resulting in falsely high (in competitive radioimmunoassays) or falsely low (with current immunometric assays) results. The cutoff level is used to determine the qualitative status of TgAb (positive or negative), and therefore, the interference of the Tg assays has been established as the functional sensitivity of the tests based on the ratio Tg-IMA to Tg-RIA. It has been shown that when the manufacturer's recommended cutoff point (used to detect thyroid autoimmunity) was used to determine the presence of TgAb, some samples showing interference were falsely classified as negative for TgAb and this lowered the limit of detection of TgAb. Functional sensitivity minimizes false negative ratings. For this reason, it is preferred to use functional sensitivity to assess the qualitative state of TgAb.

Persistent TgAb in patients without evidence of disease may be explained by different pathogenic mechanisms [20]. First, small amounts of residual normal thyroid tissue could be present. Second, the presence of TgAb could be associated with the coexistence of underlying autoimmune thyroiditis. And finally, TgAb production may be carried out by some lymphocytic memory cells [19]. The immune response may be triggered as a reaction to the inflammatory process associated with the tumorigenesis, in which Tg molecules modified by posttranslational changes behave like antigens with great immunogenicity.

Concentrations of TgAb are currently measured with immunoassays using monoclonal antibodies, and their results are traceable to the IRP 65/93 reference standard. In spite of this, the limits of quantification of the different assays and the discriminant values that each manufacturer recommends considering that a sample contains antibodies against Tg can vary by a factor of up to 200. The heterogeneity of the epitopes to which the antibodies are directed and their recognition in different TgAb assays is a known cause of variation [26]. In this regard, patients with DTC with restriction of the TgAb epitope similar to Hashimoto's disease have a higher rate of disease recurrence or persistence than in patients with nonspecific reactivity, which suggests that TgAb epitope specificities may have prognostic value [27].

The main strengths of our study are the strict follow-up during an average period of four years, the precise analytical determinations, and the homogeneity of the sample of DTC patients coming from the same geographical area of Northeastern Spain. The data presented in this study are not new in themselves but, in our opinion, they broaden the base of scientific knowledge on a controversial topic of clinical significance. In addition, thyroid autoimmunity has a multifactorial etiology with a complex interaction of environmental factors in genetically susceptible individuals. Therefore, the influence of thyroid autoimmunity in patients with thyroid cancer could be significantly different depending on the populations studied, and providing data in our area is a contribution that we consider valuable. Among the weaknesses is the retrospective design.

In conclusion, from a clinical perspective, our results show a low prevalence of TgAb in CDT patients. When TgAb is detected in a given patient, temporally close follow-up is mandatory for the definition of any TgAb temporal variation pattern, thus suggesting that not only the appearance of TgAb or significant increase in TgAb but also stable TgAb concentrations should be considered a warning sign. In these cases, active search for recurrent or persistent disease by means of highly sensitive image scans is warranted. By contrast, a significant decline in or stable persistence of TgAb levels can be considered a good prognostic sign.

## Figures and Tables

**Table 1:** Patient demographics and characteristics.

	
Sex (M/F)	15/83
Mean age, years (SD)	47 (±15)
Family history neoplasia, *n*	39
Family history thyroid disease, *n*	29
Prior neck irradiation, *n*	8
Histologic subtype, *n*	
Classical papillary	80
Tall cell	1
Sclerosing	4
Hürthle	1
Follicular	12
Mean size, cm (SD)	2.3 (±1.7)
Multifocality, *n*	33
Extrathyroidal extension, *n*	23
Lymphovascular invasion	17

TNM classification, *n*	
T1	38
T2	22
T3	28
T4	10
N1	48
M1	10

Stage, *n*	
Stage I	56
Stage II	2
Stage III	18
Stage IVA	13

**Table 2:** Characteristics of the patients with disease recurrence according to thyroglobulin autoantibodies levels during the follow-up.

TgAb	Stable				>50% increase	*de novo* appearance	
Sex (M/F)	M	F	F	F	F	F	F
Age (years)	50	73	38	52	77	70	75
Histology	Papillary	Papillary	Papillary	Papillary	Papillary	Papillary	Papillary
Initial TNM	T3N1M0	T4N1M0	T2N1M0	T4N1M1	T4N1M0	T4N1M0	T3N1M0
Diagnostic procedure	PET/TC	PET/TC	Ultrasound	PET/TC	PET/TC	Ultrasound	Ultrasound
Recurrence location	Lung M1	Lung M1	Neck N1	Lung M1	Lung M1	Neck N1	Neck N1
Recurrence time (months)	12	14	7	23	36	48	23
Tg (ng/ml)							
Initial	0.2	5.8	0.4	0.2	0.2	176	0.2
Recurrence	0.1	0.6	0.1	0.2	0.2	19	0.3
TgAb (IU/ml)							
Initial	128	3000	81	900	221	Negative	Negative
Recurrence	83	2500	71	700	2500	63	172

PET/TC: 18F-fluorodeoxyglucose positron emission tomography/computed tomography; Tg: thyroglobulin; TgAb: thyroglobulin autoantibodies; N1: lymph node metastasis; M1: distant metastasis.

## Data Availability

The clinical and analytical data used to support the findings of this study are available upon request to the corresponding author.
